# Transient Ipsilateral Hemineglect Following Brain Laser Ablation in Patient with Focal Cortical Dysplasia

**DOI:** 10.3390/neurolint16050072

**Published:** 2024-09-03

**Authors:** Georgios Ntolkeras, Fatemeh Mohammadpour Touserkani, Michelle Y. Chiu, Sanjay P. Prabhu, Scellig Stone, Alexander Rotenberg

**Affiliations:** 1 Department of Neurology, Boston Children’s Hospital, Harvard Medical School, Boston, MA 02115, USA; fatemeh.mohammadpourtouserkani@childrens.harvard.edu (F.M.T.); michelle.chiu@childrens.harvard.edu (M.Y.C.); alexander.rotenberg@childrens.harvard.edu (A.R.); 2 Department of Radiology, Boston Children’s Hospital, Harvard Medical School, Boston, MA 02115, USA; sanjay.prabhu@childrens.harvard.edu; 3 Division of Epilepsy Surgery, Department of Neurosurgery, Boston Children’s Hospital, Harvard Medical School, Boston, MA 02115, USA; scellig.stone@childrens.harvard.edu

**Keywords:** LiTT, hemineglect, epilepsy, epilepsy surgery, laser ablation

## Abstract

Sensory integration is the province of the parietal lobe. The non-dominant hemisphere is responsible for both body sides, while the dominant hemisphere is responsible for the contralateral hemi-body. Furthermore, the posterior cingulate cortex (PCC) participates in a network involved in spatial orientation, attention, and spatial and episodic memory. Laser interstitial thermotherapy (LiTT) is a minimally invasive surgery for focal drug-resistant epilepsy (DRE) that can target deeper brain regions, and thus, region-specific symptoms can emerge. Here, we present an 18-year-old right-handed male with focal DRE who experienced seizures characterized by sensations of déjà vu, staring spells, and language disruption. A comprehensive evaluation localized the seizure focus and revealed a probable focal cortical dysplasia (FCD) in the left posterior cingulate gyrus. The patient underwent uneventful LiTT of the identified lesion. Post-operatively, he developed transient ipsilateral spatial neglect and contralateral sensory loss, as well as acalculia. His sensory symptoms gradually improved after the surgery, and he remained seizure-free after the intervention for at least 10 months (until the time of this writing). This rare case of ipsilateral spatial and visual hemineglect post-LiTT in epilepsy underscores the importance of recognizing atypical neurosurgical outcomes and considering individual variations in brain anatomy and function. Understanding the dynamics of cortical connectivity and handedness, particularly in pediatric epilepsy, may be crucial in anticipating and managing neurocognitive effects following epilepsy surgery.

## 1. Introduction

Sensory integration is the province of the parietal lobe, with the non-dominant hemisphere (often right) being responsible for both sides of the body and the dominant for the contralateral hemi-body [1]. This function is shared with the insular region of the brain and, in particular, the posterior regions (granular regions) [2]. Parietal lesions, in particular injuries to the ventral frontoparietal cortex, as well as lesions to structures that are intimately connected to the parietal lobe, are often associated with visual [3] or, more broadly, spatial and sensory hemineglect [4,5,6,7,8]. Beyond the regional approach, neglect is now better understood as a dysfunction of cortical networks [8]. It reflects impairment of control of spatial attention and its reflection within an internal frame of reference in addition to deficits in reorientation, target detection, and vigilance. As a result, lesions in the non-dominant ventral frontoparietal cortex cause neglect; impair non-spatial functions, such as task-evoked activity, vigilance, and attention; and alter the functional connectivity of the associated ventral frontoparietal network. In addition, the parietal lobe is involved in other functions, such as numerical calculations. Although a multi-regional model is often proposed, acalculia has been most prominently associated with lesions of the dominant parietal lobe [9]. Lesions of this area lead to brain network reorganization in associated areas (i.e., angular gyrus, left temporoparietal and frontoparietal networks), indicating the potential of the brain to reestablish the ability to perform the task of numerical calculations by activating and utilizing secondary brain regions [10,11,12].

Up to one-third of children with epilepsy develop drug resistance and need neurosurgery that can lead to significant seizure reduction or seizure freedom [13]. The presurgical workup for patients with DRE includes the phase 1 evaluation, ensuring that multimodal data (long-term video electroencephalography (EEG), magnetic resonance imaging (MRI), magnetoencephalography (MEG), functional MRI (fMRI), positron emission tomography (PET) and neurophysiological data), are collected for the delineation of the epileptogenic zone (EZ), which is the area of the brain that is indispensable for the generation of seizures [14,15]. In complex cases, following phase 1, a phase 2 evaluation is performed, which is a detailed invasive EEG recording of the brain activity with intracranial electrodes (stereotactic EEG (sEEG) or electrocorticography (ECoG)) [16]. Based on the data acquired from the phase 1 and phase 2 evaluations, the patient can be deemed eligible for resective epilepsy surgery, laser interstitial thermotherapy (LiTT) or other interventions such as neuromodulation with a vagus nerve stimulator (VNS), deep brain stimulation (DBS) or responsive neurostimulation (RNS) [17,18]. Among the neurosurgical procedures, LiTT is a novel stereotactic approach to the surgical treatment of focal drug-resistant epilepsy (DRE). It is a minimally invasive, MR-guided method that uses implanted laser fibers to denature cellular proteins and cause controlled tissue death and can be used as a targeted therapeutic approach to patients with focal DRE with a favorable outcome-to-complications ratio [19]. Indications include focal lesions such as hypothalamic hamartomas, focal cortical dysplasia, tuberous sclerosis, mesial temporal sclerosis, and periventricular heterotopias, and due to its ability to target deeper brain regions, symptoms isolated to those regions can emerge.

Here, we present a rare case of transient ipsilateral spatial hemineglect and acalculia after left posterior cingulate laser ablation of a suspected focal cortical dysplasia (FCD) in a right-handed 18-year-old male with focal DRE.

## 2. Case Presentation

An 18-year-old right-handed male presented with seizures, refractory to medications, with onset at the age of 15 years. His seizure semiology was characterized by an aura of déjà vu, followed by staring off and language dysfunction, lasting less than 30 s. Long-term video EEG monitoring captured seizures from the left temporal region or the left posterior quadrant (cingulate gyrus and parietal lobe/precuneus), while MEG localized the epileptiform activity to his left inferior/mid temporal and left posterior parietal region. Serial 3-Tesla and research 7-Tesla MRI brain scans revealed a stable, non-enhancing lesion in the left paramedian posterior cingulate gyrus, likely consistent with FCD. Neuropsychological testing indicated left hemispheric dysfunction characterized by expressive language deficits (mainly on naming tasks) despite well-developed learning and memory skills.

Following the presurgical noninvasive evaluations, the patient underwent stereo-EEG with 15 electrodes (208 contacts) placed in the left hemisphere. Interictal findings included independent spike activity in the left posterior cingulate, hippocampus, and basal temporal region. Typical electroclinical seizures had onsets in the left mesial temporal lobe, often with simultaneous involvement of the left posterior cingulate.

**Figure 1:**
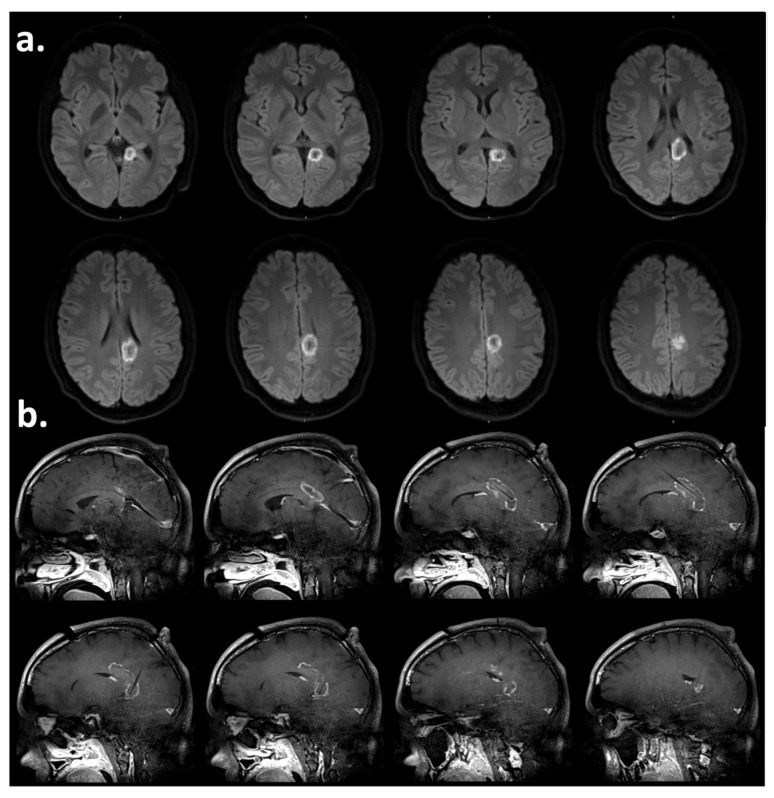
(**a**) Post-operative diffusion-weighted axial MRI and (**b**) T1-weighted contrast-enhanced sagittal magnetic resonance image showing the area of laser thermal ablation, including the posterior left cingulate gyrus and parietal isthmus, as well as a portion of the left forceps major.

Laser interstitial thermal therapy (LiTT) of the left posterior cingulate was performed instead of extensive resective surgery or neuromodulation, because there was a clear focal lesion in that area, and the stereo-EEG data supported its involvement in his epileptogenic network. For the procedure, left frontal and parietal trajectories were planned such that two 10 mm diffusing tip Visualase (Medtronic Inc., Minneapolis, MN, USA) fibers would be able to perform a substantive ablation of the target region. Laser fibers were inserted under general anesthesia using a stereotactic frame and robotic assistance along with intraoperative MRI confirmation of insertion accuracy and lack of intracranial hemorrhage. LiTT was then performed using live MR thermography imaging. Lesioning was performed on one laser at a time by first activating the laser at a low setting to confirm the position of the diffusing tip on thermography images and subsequently increasing the energy of the laser to that which would create a lesion. During the procedure, there were no hemodynamic changes. Finally, diffusion-weighted images were acquired, which confirmed that the software-predicted lesion volume had been achieved and a total ablation of the desired target was accomplished. The total anesthesia time was five hours and thirty minutes. The anesthetics that were used were vecuronium, propofol, dexmedetomidine, and midazolam, while the analgesics that were used were fentanyl and hydromorphone. There was no evidence of injury/ischemia or infarction outside of the intended ablation zones. The procedure was uncomplicated, and intraoperative MRI findings were as expected, showing an L-shaped ablation zone with an approximately 66 mm anteroposterior plane and 15 mm diameter with peripheral enhancement and diffusion restriction with no abnormal findings outside the expected ablation zone (Figure 1).

**Figure 2:**
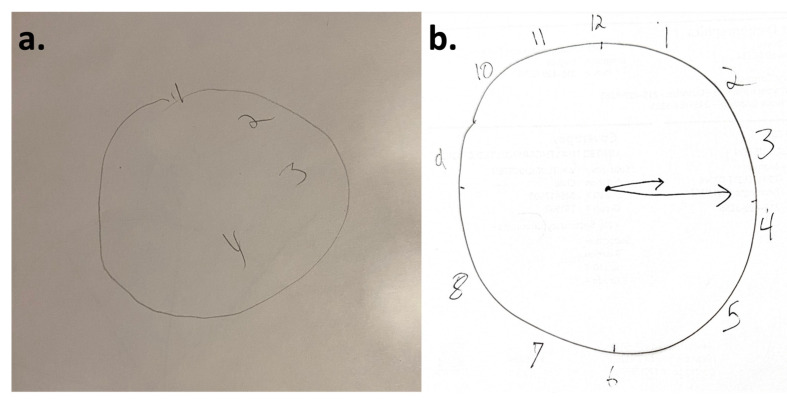
(**a**) The classic clock as drawn by the patient in the immediate post-operative period. An asymmetrical large right side can be appreciated, as well as numerical markings completed only on the right side of the clock. The patient appeared remarkably confused when he reached number four and aborted the task considering it completed. (**b**) The classic clock drawn by the patient at his 10-month post-operative clinic visit, indicating no signs of hemineglect.

Post-operatively, the patient exhibited transient ipsilateral sensory neglect and contralateral partial sensory loss. His initial post-operative physical examination revealed decreased sensation to light touch, pinprick, and temperature in the right upper and lower extremities, left-sided agraphesthesia, and extinction to double simultaneous (tactile and visual) stimulation on the left. He also displayed difficulty with the clock drawing task, which was performed eight hours after the procedure was completed, only being able to draw numbers on the right side of the clock and being confused when reaching the midline (Figure 2a). His motor and strength exam remained without impairments throughout his hospitalization, while his sensory symptoms and hemineglect improved by the time of his discharge, three days after surgery. During the recovery period after the surgery, the patient used acetaminophen for analgesia.

Post-surgery, at the 1-month follow-up visit, the patient saw notable improvements, though with lingering difficulties. His right-sided sensation and coordination had almost returned to presurgery levels, with the exception of persistent yet improving impairments in his left hand (coordination impairment noticed in fine tasks such as dribbling a basketball, with no accompanying weakness). Academically, as an accomplished math student, he initially struggled with numerical calculations, temporal judgments, and sequential event recall after his surgery, symptoms which have steadily improved. He graduated from high school with good grades and is entering his freshman year at university at the time of writing, 10 months after his surgery. The patient has remained seizure-free since surgery at the 10-month follow-up visit, during which he displayed no symptoms of hemineglect, sensory or spatial (Figure 2b), and he had no difficulties with calculations or sequential event recall either on his mental status neurologic exam. On the same day, he had a routine EEG that did not reveal any seizures, background abnormalities, or interictal epileptiform discharges while, since the surgery, he has been on stable doses of his anti-seizure medications (levetiracetam and lamotrigine). His repeat 3-Tesla brain MRI at the same follow-up visit was also stable and showed expected post-surgical changes related to the laser ablation within the left cingulate gyrus and the parietal isthmus.

## 3. Discussion

This is a case of an 18-year-old right-handed male with focal DRE, whose clinical seizure semiology was characterized by an aura of déjà vu, suggesting the involvement of mesial–temporal regions, language impairment, and staring with no responsiveness, that localized to the dominant (left) hemisphere [20]. The next step in the evaluation of a patient with DRE, with a suspected focal mechanism of seizure onset, is often to investigate their eligibility for epilepsy surgery, which can offer seizure freedom or better seizure control [13]. This process starts with a phase 1 evaluation that includes long-term video-EEG monitoring [21] and more advanced neurophysiological techniques, such as MEG [22], that can offer diagnostic clarity and accuracy in localizing the epileptogenic zone (EZ), the area of the brain that is indispensable for the generation of seizures [14]. Finally, anatomical neuroimaging with a brain MRI is critical for the identification of structural abnormalities that may underlie seizure disorders, such as malformations of cortical development (i.e., FCD, polymicrogyria, and other disorders), with an identifiable abnormality being detected in about 85% of the cases [23]. Our patient had 3- and 7-Tesla MRI that were significant for an area of signal abnormality, in the left paramedian posterior cingulate gyrus that could represent an area of FCD. This concluded the phase 1 presurgical evaluation that captured seizures arising from the left temporal region or the left posterior quadrant, with the MEG localizing interictal epileptiform activity to the same region and the MRI pointing to the left paramedian posterior cingulate gyrus.

In cases where the delineation of the EZ is complicated due to non-localizing or incongruent findings of the phase 1 evaluation or due to complex brain network involving multiple areas or high-risk areas of the eloquent cortex, invasive EEG recordings are applied to aid with increasing the precision of the presurgical planning by mapping the epileptogenic network, in a process called phase 2 evaluation [16]. Invasive EEG recordings include stereotactic EEG (sEEG) with in-depth electrodes and electrocorticography (EGoG) with subdural electrodes. Despite both techniques being effective in delineating the EZ, recent studies indicate that sEEG is more appropriate for identifying deeper sources and has an overall lower complication rate [24]. In our patient’s case, the identification of a complex epileptogenic network potentially including both the left posterior cingulate and left mesial temporal structures with the eloquent cortex, which could have an impact on memory (the dominant temporal lobe), led to the decision for a phase 2 evaluation, and 15 electrodes with a total 208 contacts were implanted. Finally, the decision was made for an LiTT ablation of the FCD lesion of the posterior cingulate gyrus as an initial step, given its focality and participation in the epileptogenic network, with responsive neurostimulation (RNS) being a secondary option, which was not pursued at that time [25]. The surgery was completed successfully without intraoperative complications.

Post-operatively, the patient developed transient ipsilateral spatial neglect, contralateral sensory loss, and acalculia. The intraoperative MRI delineated the ablation in the left posterior cingulate and splenium and showed no operative complications or lesions outside the expected ablation area (Figure 1). These findings potentially explain the right-sided sensory disturbances, given the potential irritation of the white matter tracts as they run into the contralateral sensory cortex. However, the diminished sensation on the left side, as well as the left visuospatial hemineglect, remained unexplained by the structural brain MRI findings, since there were no right-sided interventions or approaches with this surgery (Figure 2a). This opens a window to the potential role of areas such as the left posterior cingulate region in spatial orientation and sensory perception. In addition, his symptoms of acalculia would also localize in the same area of the dominant (left) parietal lobe, which was targeted by the LiTT [9]. As reported by Tao and Rapp, patients with left frontoparietal strokes displayed impaired functional connectivity with functional MRI studies, with abnormally low interhemispheric connectivity and increased intrahemispheric connectivity. Recovery was associated with the normalization of those findings with increases in connectivity in both the left and right dorsal frontoparietal homotopic regions [26]. This indicates the involvement of both hemispheres in the recovery process of unilateral strokes, potentially leading to symptoms that would otherwise localize to a primary lesion of the right hemisphere, to transiently emerge potentially as part of the brain network reorganization during the recovery phase of his brain surgical ablation of the left posterior parietal lobe. In addition, studies of functional connectivity with MEG demonstrated a functional balance between homologous somatosensory areas in the hemispheres, with the younger patients having lower variability compared to older individuals supporting the bi-hemispheric network involvement in the recovery phase [27]. As a result, our patient’s presentation possibly reflects the more recent descriptions of hemineglect as a phenomenon of brain network disruption [8].

Furthermore, an explanation of this patient’s clinical symptoms may be associated with his epilepsy diagnosis and underlying pathology. Focal cortical dysplasia is associated with disruptions in the mTOR pathway, while somatic mutations along the neural migration can cause different extents of cortical malformation, with mutations happening earlier in the process, leading to more extensive lesions [28,29]. Handedness and brain asymmetry are also dynamic processes that take place in early childhood [30,31]. This process may be prolonged or altered in several conditions, including epilepsy, which can affect brain development and connectivity, as shown by studies demonstrating a delay in the white matter volume increase and, thus, the anatomical connections between brain regions in patients with epilepsy, as well as a higher incidence of development of atypical handedness in patients with epilepsy compared to the general population [32,33]. In addition, animal and human studies have shown that epilepsy, as a network disease, can alter the body’s representation of the somatosensory cortex [34,35]. In mouse models, increased functional connectivity has been shown between the somatosensory and cingulate cortex [36], and focal epilepsy was shown to disrupt sensory-evoked neurovascular coupling in both ipsilateral and contralateral sensory cortices indicating a disease-induced alteration of the body’s representation in the somatosensory cortex [37]. As a result, a possible explanation of this patient presentation could be that his FCD and associated epilepsy disrupted the lateralization process in his development and led to brain network reorganization, leading to an atypical presentation of ipsilateral spatial perception deficits exacerbated by partial network disruption due to operative irritation and potential damage adjacent to the cingulate gyrus regions, such as white matter tracts entering the corpus callosum, that eventually improved given that the ablation was of limited extent and was unlikely to lead to persistent hemispheric isolation [38].

This is a rare case of an 18-year-old right-handed male with DRE who developed transient ipsilateral visual, tactile, and spatial hemineglect after having LiTT in the left posterior cingulate area with no intraoperative surgical complications and with his post-operative MRI only showing expected surgical changes. This case offers valuable insight into the potential mechanisms of hemineglect and cortical organization in patients with brain injury, and in particular in patients with focal epilepsy. Finally, it contributes to the literature by discussing the presentation of hemineglect in a young individual with a unique phenotype and brain dynamics.

## 4. Limitations

This case’s limitations include a lack of complete neuropsychiatric evaluation testing during the acute post-operative phase, as this is not part of the clinical practice in acute post-operative care, which would have complemented our neurologic examination and would have provided further insight into the patient’s clinical presentation. This limitation is common in clinical case reports and could be addressed in a controlled prospective study design. In addition, functional MRI or high-density EEG recordings in the acute post-operative phase would allow for in-depth analysis of the functional connectivity of the brain along with other measures of brain dynamics, and serial recordings would allow for tracking of the neurodegenerative process and network reorganization during the recovery phase, although those studies were not available given the retrospective nature of this case report. Finally, having a larger number of cases with similar clinical presentations would allow for a systematic analysis of this clinical syndrome.

## 5. Conclusions

This case report sheds light on a unique presentation of transient ipsilateral spatial hemineglect following LiTT in the left (dominant) posterior cingulate area in a young patient with focal DRE. It emphasizes the need to consider individual variations in cerebral function and lateralization, especially when evaluating neurosurgical outcomes influenced by pre-existing focal epileptogenic lesions.

## Data Availability

The original contributions presented in the study are included in the article, further inquiries can be directed to the corresponding authors.
